# Minocycline synergizes with corticosteroids in reducing colitis severity in mice via the modulation of pro-inflammatory molecules

**DOI:** 10.3389/fphar.2023.1252174

**Published:** 2023-11-16

**Authors:** Maitham A. Khajah, Sanaa Hawai, Ahmad Barakat, Aisha Albaloushi, Maha Alkharji, Willias Masocha

**Affiliations:** College of Pharmacy, Kuwait University, Kuwait City, Kuwait

**Keywords:** colitis, DSS, minocycline, methylprednisolone, signaling pathway

## Abstract

**Background:** A few studies have highlighted the anti-inflammatory properties of minocycline in reducing colitis severity in mice, but its molecular mechanism is not fully understood. The aim of this study was to determine the anti-inflammatory properties of minocycline and the expression/activity profiles of molecules involved in pro-inflammatory signaling cascades, cytokines, and molecules involved in the apoptotic machinery. The synergistic effect between minocycline and corticosteroids was also evaluated.

**Methods:** The effects of various treatment approaches were determined in mice using the dextran sulfate sodium (DSS) colitis model at gross and microscopic levels. The expression/activity profiles of various pro- or anti-inflammatory molecules were determined using Western blotting and polymerase chain reaction (PCR).

**Results:** Minocycline treatment significantly reduced colitis severity using prophylactic and treatment approaches and produced a synergistic effect with budesonide and methylprednisolone in reducing the active state of colitis. This was mediated in part through reduced colonic expression/activity of pro-inflammatory molecules, cytokines, proteins involved in the apoptotic machinery, and increased expression of the anti-inflammatory cytokine IL-10.

**Conclusion:** Minocycline synergizes with corticosteroids to reduce colitis severity, which could reduce their dose-dependent side effects and treatment cost. The reduction in colitis severity was achieved by modulating the expression/activity profiles of various pro- and anti-inflammatory signaling molecules, cytokines, and molecules involved in the apoptotic machinery.

## Introduction

Inflammatory bowel disease (IBD) is a chronic inflammatory condition affecting the gastrointestinal tract (GIT) and may also contribute to the development of various extra-intestinal complications in affected individuals. It primarily comprises two major conditions: Crohn’s disease (CD) and ulcerative colitis (UC) (Hanauer, 2006). The prevalence rates of IBD in Europe and North America range from 8 to 214 per 100,000 for CD and 21 to 246 per 100,000 for UC (Feuerstein et al., 2014). The incidence has been increasing in newly industrialized countries in Asia, South America, and the Middle East (Ng et al., 2018). Crohn’s disease mainly manifests as a patchy pattern of an inflammatory process affecting the whole GIT, although it is more common in the terminal ileum and the right colon with enhanced production of T helper 1 (T_H_1) cytokines such as interleukin (IL)-12, IL-2, IL-1β, tumor necrosis factor alpha (TNF-α), and interferon gamma (IFN-γ) (Boyapati et al., 2015). With regards to UC, inflammation is superficial with a diffuse pattern affecting mainly the colon, with enhanced production of T_H_2 cytokines such as IL-4, -5, -6, and -13 (Xavier and Podolsky, 2007). Although there are many therapeutic options currently available for the treatment of IBD, many of them fail to induce and maintain remission in approximately 30% of patients (van Deventer, 2002) and are associated with significant side effect profiles. Moreover, a significant proportion of patients are either steroid-resistant (20%) or steroid-dependent (30%), while the remaining 50% are neither resistant to nor dependent on steroid treatment (Farrell and Kelleher, 2003). Biological therapies can be used for the treatment of steroid-refractory patients, but they have many drawbacks, such as restrictions on non-oral routes of administration that require multiple hospital visits, a high cost of treatment, the immunogenicity of the product, which reduces its efficacy in long-term therapy regimens, and serious side effect profiles (van Deventer, 2002). Therefore, there is a need to find new therapeutic targets and agents that may offer alternatives to treat this chronic inflammatory condition. In a recent study, a combination regimen of one antibiotic with corticosteroids significantly induced disease remission in patients with active UC (Uehara et al., 2010). The findings of this study suggest that targeting the bacterial components and the immune response at the same time might be a better approach to the management of the disease.

Minocycline is a widely used second-generation, semi-synthetic tetracycline with antibacterial, antiviral, and anti-inflammatory properties (Garrido-Mesa et al., 2013a). It is considered a relatively safe antibiotic with broad-spectrum properties against many Gram-negative and Gram-positive bacteria like *E. coli*, *P. aeruginosa*, *and B. subtilis*, and *S. aureus*, respectively (Chen et al., 2000). Its mechanism of action is through the inhibition of protein synthesis by binding to the bacterial 30S ribosomal subunit. Interestingly, minocycline also possesses other biological actions beyond its antibacterial properties, such as anti-inflammatory, anti-apoptotic, and immunosuppressive properties, that are widely useful in various pathological conditions, such as acne vulgaris, rheumatoid arthritis, periodontitis, asthma, Parkinson’s disease, neural ischemic damage, and Huntington’s disease (Garrido-Mesa et al., 2013a).

Currently, there are a few studies that have described the role of minocycline in animal models of colitis. However, its molecular mechanism of action has not been fully elucidated as yet. Minocycline treatment reduced colitis severity (by using the treatment but not the prophylactic approach) in mouse models of chemically induced colitis, i.e., the dextran sulfate sodium (DSS) and trinitrobenzene sulfonic acid (TNBS) colitis models (Garrido-Mesa et al., 2013b). A previous study using the DSS model reported that minocycline (both prophylactic and treatment approaches) significantly reduced colitis severity at macroscopic and histological levels (Huang et al., 2009). The main aim of the present study was to determine the synergistic effect between minocycline and current clinically available treatment options, i.e., corticosteroids, for colitis. Another aim was to determine the contribution of the various compounds in the inflammatory response by examining the colonic expression/activity profiles of molecules involved in the pro-inflammatory signaling cascades, cytokines, and molecules involved in the apoptotic machinery. Studying the effects of the combination of minocycline with corticosteroids would be of clinical importance since this could improve therapeutic outcomes, reduce the doses of each drug, and thus reduce their dose-dependent side effects. Prednisolone/methylprednisolone and budesonide are widely used corticosteroids in IBD patients and animal models of colitis; therefore, they were used in this study as two agents from this category of drugs. In an animal model of colitis, prednisolone (2–5 mg/kg) was shown to reduce colitis severity at macroscopic and histological levels (Nirmal et al., 2013; Myrelid et al., 2015). Furthermore, treatment with methylprednisolone (0.4 mg/day) reduced the colonic inflammatory score and the amount of fecal bacteria in the colon of rats with DSS-induced colitis (Wang et al., 2011). Budesonide was also shown to reduce colitis severity in mice with DSS- and acetic acid-induced colitis, partly through reducing the expression profile of various mediators/cytokines such as myeloperoxidase, nitric oxide, TNF-α, IL-6, cyclooxygenase-2 (COX-2), and inducible nitric oxide synthase (iNOS) (Ali et al., 2014; Mishra et al., 2023; Seoudi et al., 2023; Xian et al., 2023; Yang et al., 2023).

Herein, we showed experimental evidence for the anti-inflammatory properties of minocycline using both the prophylactic and treatment approaches, and its ability to synergize with two corticosteroids, methylprednisolone and budesonide, in an established colitis setting (using the treatment approach). This was mediated in part through reduced colonic expression/activity of pro-inflammatory molecules, cytokines, proteins involved in the apoptotic machinery, and increased expression of the anti-inflammatory cytokine IL-10.

## Materials and methods

### Animals

The animals used in this study were female BALB/c mice (aged 6–10 weeks old with a mean weight of 20 g) that were supplied by the Animal Resource Center of the Health Sciences Center at Kuwait University. The mice were kept under standard conditions, including controlled temperature (25°C ± 1°C), a 12-h light–dark cycle, and free access to food and drinking water. All experiments were approved by the Animal Care Committee at Kuwait University Health Sciences Center and conformed to their rules and regulations as described previously (protocol approval number: P11613PT01) (Khajah et al., 2016a).

### Dextran sulfate sodium colitis model

Colitis was induced in mice by mixing DSS polymers with the drinking water (3.5% w/v, Cat # 160110, MP Biomedicals) given *ad libitum* (Wirtz et al., 2007; Khajah et al., 2016a). Control, untreated (UT) mice received tap water. Daily weight changes were determined as a loss of baseline body weight. Colon length and maximal bowel thickness (in millimeters) were also determined (Khajah et al., 2016a). Two approaches were used in this study: a) prophylactic and b) treatment. For the prophylactic approach, mice received daily intra-peritoneal (i.p) injections of minocycline (10–40 mg/kg, Cat# M9511, Sigma) or vehicle (saline) along with DSS administration for 5 days. For the treatment approach, mice received daily DSS administration for 4 days (to first establish a state of active colitis), followed by daily i.p injections of minocycline (1–40 mg/kg), budesonide (0.1–100 μg/kg, Cat # 01901680540,365, AstraZeneca), methylprednisolone (0.01–10 mg/kg, Cat #P6004, Sigma), a combination regimen of minocycline plus either budesonide or methyl prednisolone, or vehicle (saline) for 3 days (without DSS administration). After that, mice were sacrificed (on day 5 after the first administration of DSS for the prophylactic approach and on day 7 after the first administration of DSS for the treatment approach), and the severity of colitis was determined by macroscopic (gross) and microscopic (histologic) assessments.

### Macroscopic assessment of colitis severity

The macroscopic assessment for colitis severity in all treatment groups was performed as previously described (Khajah et al., 2016a). The macroscopic assessment data are presented in tables as the percentage (%) of mice in each treatment group, showing various features of colitis (edema, erythema, diarrhea, blood in stool, anorectal bleeding, and adhesion).

### Microscopic assessment of colitis severity

For the microscopic assessment of colitis severity, formalin-fixed colon tissues (the whole colon) were processed and (blindly) scored by two observers using a standard semi-quantitative histology scoring system, as previously described (Khajah et al., 2016a).

### Western blotting

The colonic (descending part) protein expression of phospho/total ERK1/2 (Cat # 4370 and 9102), p38 MAPK (Cat # 9216), phospho-AKT (Cat # 9271), AKT (Cat # 9272), COX-2 (Cat # 4842), SRC (Cat # 5473), BAK (Cat # 12105), caspase-3 (Cat # 9662), the granulocyte marker LY6G (Cat # 87048), E-cadherin (Cat # 3195), and actin (loading control, Cat # 8457) (all from Cell Signaling, United States, 1:1000 dilution) were determined from all groups of mice by Western blotting, as described previously (Khajah et al., 2016b). In brief, colonic tissue was snap-frozen in liquid nitrogen and stored at −80°C until it was needed for further experiments. The colonic tissue samples were pulverized while frozen and then transferred to lysis buffer (pH 7.6) containing 10 mM Tris-base, 140 mM NaCl, 10 mM Na_4_P_2_O_7_, 1 mM NaF, 1 mM CaCl_2_, 1 mM MgCl_2_, 10% glycerol, 1% NP40 (Tergitol-type NP-40 and nonyl phenoxypolyethoxylethanol), 2 mM Na_3_VO_4_, and a protease inhibitor cocktail. The tissues were homogenized using Polytron PT 4000 (Switzerland), and the samples were left to lyse completely by incubating them on an ice-cold shaker for 30 min. Subsequently, they were centrifuged at 14,000 rpm for 20 min at 4°C, and the supernatants were collected. Protein concentrations in the supernatants were measured using a Lowry/Bradford protein assay. Aliquots containing equal amounts of protein (10 µg) were subjected to gel electrophoresis and transferred onto nitrocellulose/PVDF membranes. Membranes were then subjected to BSA (2%–4%) for 1 h and then incubated with the appropriate antibodies and subsequently with appropriate secondary antibodies conjugated to horseradish peroxidase. Immunoreactive bands were detected with the SuperSignal chemiluminescent substrate (Pierce, United Kingdom) using Kodak autoradiography film (G.R.I., Rayne, United Kingdom).

### Real-time reverse transcription–polymerase chain reaction (RT-PCR)

Real-time RT-PCR was used to quantify the mRNA of the cytokines, TNF-α, IL-1β, and IL-10, in the colon and blood. The descending part of the colon was dissected and snap-frozen in liquid nitrogen. On the day of RNA extraction, the descending part of the colon was homogenized in 1.5 mL of lysis buffer from the RNeasy^®^ Mini Kit, containing 15 μL of β-mercaptoethanol. Blood was extracted from the mice by cardiac puncture and added to the RNAprotect Animal Blood Tube (100 μL). The tube with blood was inverted eight to ten times and left standing at 15–25°C for 2 h. The contents were transferred to a 1.5-mL Eppendorf tube. This tube was centrifuged for 3 min at 5,000 × g at 20°C, the supernatant was discarded, and 1 mL of RNase-free water was added and vortexed with the pellet; this was centrifuged for 3 min at 5,000 × g at 20°C, and the supernatant was discarded. Buffer RSB (240 μL) from the RNeasy^®^ Protect Animal Blood Kit was added to the pellet, vortexed until dissolved, and used for RNA extraction. Total RNA was extracted from homogenized colon samples and prepared into blood samples using the RNeasy^®^ Mini Kit and RNeasy^®^ Protect Animal Blood Kit, respectively, following the manufacturer’s protocols, and quantified by spectrometry. The total RNA (500 ng) was used to synthesize cDNA, as previously described (Masocha, 2009). Real-time RT-PCR amplification was performed in triplicate using an ABI Prism^®^ 7500 Sequence Detection System (Applied Biosystems). A measure of 1 µL of cDNA was added to a 19 µL reaction mixture containing 1× Platinum^®^ SYBR^®^ Green qPCR Supermix-UDG, ROX dye, and forward and reverse primers (125–500 nM), and amplified as previously described (Masocha, 2009). The primer sequences that were used (Table 1) were extracted from previous publications from other laboratories (Li et al., 2017) and our laboratory (Parvathy and Masocha, 2013), checked for specificity by BLAST in the GenBank database, and ordered from Invitrogen by Life Technologies. The housekeeping genes 18S ribosomal RNA (*18s*) and cyclophilin A (*Ppia*) were used to normalize the amount of transcripts in individual animal samples (ΔCT; *n* = 3 to 5 per group for blood and *n* = 7 to 19 for colon). The relative amount of target gene mRNA in the blood and colons was calculated using the previously described 2^−ΔΔCT^ method (Livak and Schmittgen, 2001).

**TABLE 1:** Primer sequences of genes used in real-time RT-PCR.

Name	Polarity	Sequence 5′to 3′
*18s*	Sense	CGGCTACCACATCCAAGGAA
	Anti-sense	GCTGGAATTACCGCGGCT
*Ppia*	Sense	GCTTTTCGCCGCTTGCT
	Anti-sense	CTCGTCATCGGCCGTGAT
*Il1b*	Sense	TGGTGTGTACGTTCCCATT
	Anti-sense	CAGCAGAGGCTTTTTTGTTG
*Tnf*	Sense	GGCTGCCCCGACTACGT
	Anti-sense	GACTTTCTCCTGGTATGAGATAGCAAA
*Il10*	Sense	CAGCCGGGAAGACAATAACTG
	Anti-sense	CCGCAGCTCTAGGAGCATGT

### Statistical analysis

Data were analyzed using GraphPad InStat and Prism software programs (California, United States). Differences between groups were assessed using the Student unpaired *t*-test, Mann–Whitney test, one-way ANOVA followed by a Bonferroni *post hoc* test, or Kruskal–Wallis test, followed by Dunn’s multiple comparisons test, with *p* < 0.05 being regarded as significant.

## Results

### Effect of prophylactic treatment with minocycline on colitis severity

**FIGURE 1:**
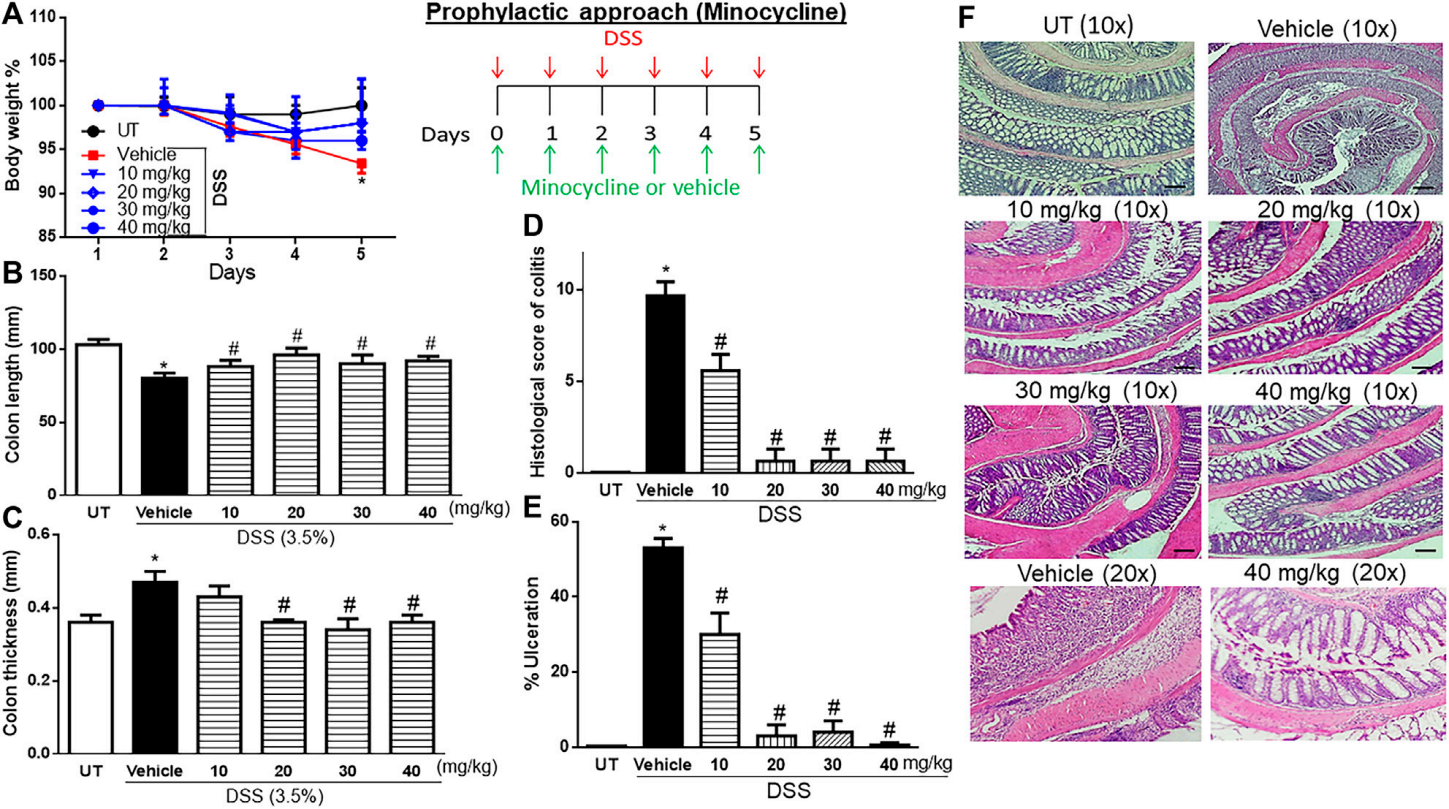
Effect of prophylactic treatment with minocycline on colitis severity at the macroscopic and microscopic levels. **(A)** Percentage of body weight changes in DSS-treated mice receiving vehicle and minocycline-treated mice at various doses compared to untreated (UT) mice. Colon length **(B)**, thickness **(C)**, histological assessment of colitis severity **(D)**, and the percentage of ulceration in the whole colon section **(E)** were determined in mice receiving DSS and either vehicle (solid bars) or various doses of minocycline (hatched bars) and in the UT healthy mice (open bars). Histobars represent means ± SEM for seven mice in each group. Asterisks denote a significant difference from UT mice with *p* < 0.05 (*), and # denotes significant difference from DSS/vehicle-treated mice with *p* < 0.05. **(F)** Illustration of colon sections taken from various groups (×10 and ×20 magnifications; bars represent 150 μm).

A significant drop in body weight from the baseline level (7%) was observed in mice that received DSS plus vehicle compared to UT healthy mice (that received tap water only) (*p* < 0.05; Figure 1A). Treatment with minocycline (10–40 mg/kg) prevented the DSS-induced weight loss, and the groups treated with minocycline had body weight similar to that of the UT group. A significant decrease in colon length (Figure 1B) and increase in colon thickness (Figure 1C) were observed in mice treated with DSS plus vehicle compared to UT mice (*p* < 0.05). Treatment with minocycline (20–40 mg/kg) prevented the DSS-induced changes in colon length and width, and the groups treated with minocycline had a colon length and width similar to those of UT mice. No signs of inflammation at the gross level were observed in UT healthy mice (Table 2). DSS plus vehicle administration resulted in edema (100%), erythema (28%), diarrhea (57%), anorectal bleeding (28%), and adhesions (100%). Minocycline treatment at doses of 30–40 mg/kg significantly reduced the presence of edema. Treatment with minocycline at doses of 20–40 mg/kg prevented the development of DSS-induced erythema, diarrhea, anorectal bleeding, and adhesions. Administration of DSS caused a significant increase in the histological score of colitis (Figure 1D) and percentage of ulceration in the whole colon (Figure 1E) compared to UT (*p* < 0.05). Treatment with DSS plus minocycline (at all doses, but specifically with 20–40 mg/kg) resulted in a significantly reduced histological score of colitis and percentage of ulceration compared to treatment with DSS plus vehicle. Figure 1F shows examples of colon sections taken from UT, DSS plus vehicle, and DSS plus minocycline-treated mice. UT mice have normal colon architecture. DSS plus vehicle resulted in significant destruction of mucosal architecture, immune cell infiltration, submucosal edema, and muscle wall thickness. Treatment with DSS plus minocycline prevented the destruction of the mucosal architecture and muscle thickening, and significantly reduced the degree of immune cell infiltration and submucosal edema.

**TABLE 2:** Effect of prophylactic treatment with minocycline on the macroscopic scores of colitis severity.

Parameter (%)	Edema	Erythema	Diarrhea	Blood in stool	Anorectal bleeding	Adhesion
UT	0	0	0	0	0	0
Vehicle	100 *	28 *	57 *	0	28 *	100 *
10 mg/kg	85 *	57 *	42 *	0	33 *	42 *#
20 mg/kg	83 *	0 #	0 #	0	0 #	0 #
30 mg/kg	33 *#	0 #	0 #	0	0 #	0 #
40 mg/kg	33 *#	0 #	0 #	0	0 #	0 #

Colitis severity was assessed in UT mice (received tap water only) and mice treated with DSS for 5 days, along with daily i. p administration of minocycline or vehicle (PBS) (*n* = 7 per group). * denotes a significant difference from UT mice; # denotes a significant difference from DSS/vehicle-treated mice.

### Effect of treatment with minocycline on the severity of already established colitis

**FIGURE 2:**
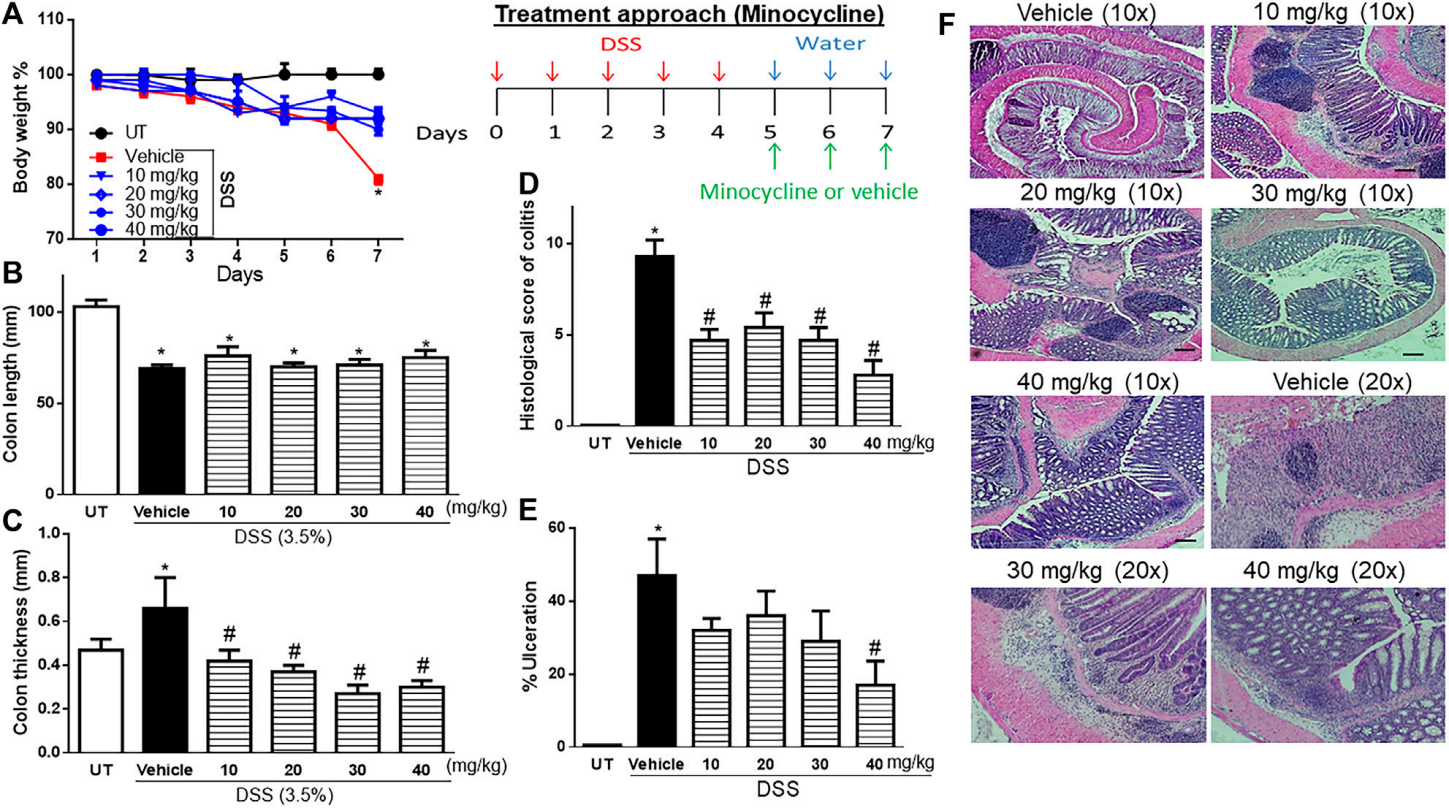
Effect of therapeutic treatment with minocycline on colitis severity at the macroscopic and microscopic levels. **(A)** Percentage of body weight changes in DSS-treated mice receiving vehicle and minocycline-treated mice at various doses compared to untreated (UT) mice. Colon length **(B)**, thickness **(C)**, histological assessment of colitis severity **(D)**, and the percentage of ulceration in the whole colon section **(E)** were determined in mice receiving DSS and either vehicle (solid bars) or various doses of minocycline (hatched bars) and in the UT healthy mice (open bars). Histobars represent means ± SEM for seven mice in each group. Asterisks denote a significant difference from UT mice with *p* < 0.05 (*), and # denotes a significant difference from DSS/vehicle-treated mice with *p* < 0.05. **(F)** Illustration of colon sections taken from various groups (×10 and ×20 magnifications; bars represent 150 μm).

Treatment with minocycline (10–40 mg/kg) reversed both the DSS-induced drop in body weight (*p* < 0.05; Figure 2A) and increase in colon thickness (*p* < 0.05; Figure 2C) but had no significant effects on colon length (Figure 2B). Minocycline treatment at doses of 20–30 mg/kg significantly reduced DSS-induced edema, erythema, diarrhea, blood in stool, anorectal bleeding, and adhesions (Table 3). These signs of DSS-induced inflammation were absent in mice treated with minocycline at a dose of 40 mg/kg. Minocycline treatment (at all doses) significantly reduced the DSS-induced histological score of colitis by 50%–60% (Figure 2D), and a minocycline dose of 40 mg/kg reduced the percentage of ulceration by 60% (Figure 2E). Figure 2F shows examples of colon sections taken from different treatment groups. Minocycline treatment (specifically at 30–40 mg/kg doses) significantly restored mucosal architecture and muscle thickening, with a significant reduction in the degree of immune cell infiltration and submucosal edema.

**TABLE 3:** Effect of therapeutic treatment with minocycline on the macroscopic scores of colitis severity.

Parameter (%)	Edema	Erythema	Diarrhea	Blood in stool	Anorectal bleeding	Adhesion
UT	0	0	0	0	0	0
Vehicle	100 *	71 *	71 *	50*	100 *	85 *
10 mg/kg	100 *	0 #	71 *	0	57 *#	71 *
20 mg/kg	66 *#	0 #	50 *#	0	50 *#	66 *#
30 mg/kg	33 *#	0 #	50 *#	0	0 #	50 *#
40 mg/kg	0 #	0 #	0 #	0	0 #	0 #

Colitis severity was assessed in UT mice and mice treated with DSS for 4 days, followed by 3 days of daily i. p administration of minocycline or vehicle (PBS) (without DSS treatment) (*n* = 7 per group). * denotes a significant difference from UT mice; # denotes a significant difference from DSS/vehicle-treated mice.

### Effect of treatment with budesonide on the severity of already established colitis

**FIGURE 3:**
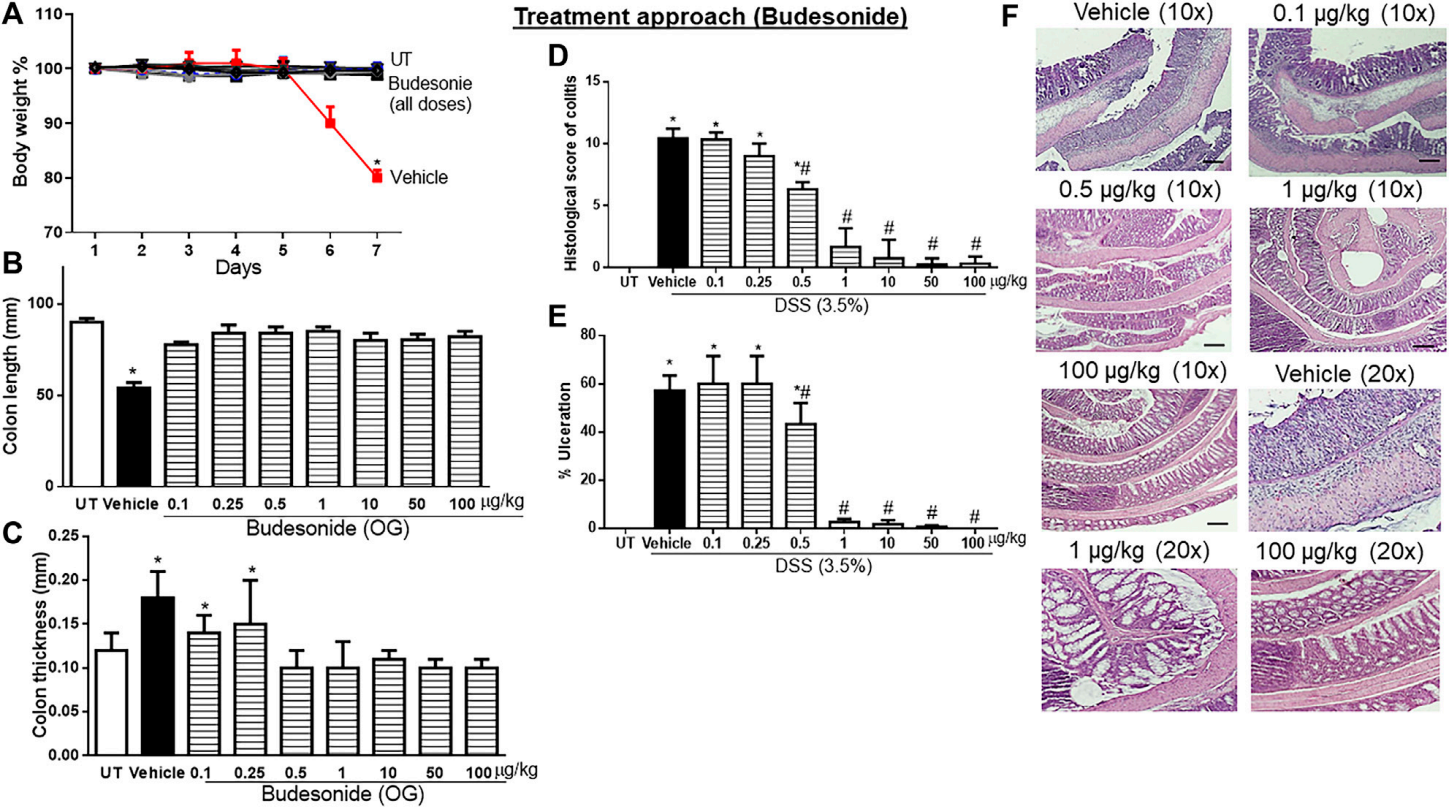
Effect of therapeutic treatment with budesonide on colitis severity at the macroscopic and microscopic levels. **(A)** Percentage of body weight changes in DSS-treated mice receiving vehicle and budesonide-treated mice at various doses compared to untreated (UT) mice. Colon length **(B)**, thickness **(C)**, histological assessment of colitis severity **(D)**, and the percentage of ulceration in the whole colon section **(E)** were determined in mice receiving DSS and either vehicle (solid bars) or various doses of budesonide (hatched bars) and in the UT healthy mice (open bars). Histobars represent means ± SEM for seven mice in each group. Asterisks denote a significant difference from UT mice with *p* < 0.05 (*), and # denotes a significant difference from DSS/vehicle-treated mice with *p* < 0.05. **(F)** Illustration of colon sections taken from various groups (×10 and ×20 magnifications; bars represent 150 μm). OG, oral gavage.

Budesonide treatment (at all doses) reversed both the DSS-induced drop in body weight (*p* < 0.05; Figure 3A) and decrease in colon length (*p* < 0.05; Figure 3B). Treatment with budesonide at doses from 0.5 to 100 μg/kg reversed the DSS-induced increase in colon thickness (*p* < 0.05; Figure 3C). Budesonide treatment at doses of 0.5–1 μg/kg significantly decreased DSS-induced edema, erythema, diarrhea, blood in stool, anorectal bleeding, and adhesions (Table 4), and these signs of inflammation were absent at budesonide doses of 10–100 μg/kg. Budesonide treatment significantly reduced the DSS-induced histological score of colitis and percentage of ulceration by approximately 50% at a dose of 0.5 μg/kg and by 80%–98% at higher doses of 10–100 μg/kg (Figures 3D, E). Figure 3F shows examples of colon sections taken from different treatment groups. Treatment with budesonide at doses of 1–100 μg/kg significantly restored all these parameters.

**TABLE 4:** Effect of therapeutic treatment with budesonide on the macroscopic scores of colitis severity.

Parameter (%)	Edema	Erythema	Diarrhea	Blood in stool	Anorectal bleeding	Adhesion
UT	0	0	0	0	0	0
Vehicle	100 *	100 *	100 *	100 *	100 *	100 *
0.1 µ/kg	100 *	100 *	100 *	100 *	100 *	66 *
0.25 µ/kg	66 *	0 #	0 #	0 #	66 *	66 *
0.5 µ/kg	33 #	0 #	0 #	0 #	0 #	33 #
1 µ/kg	33 #	0 #	0 #	0 #	0 #	33 #
10 µ/kg	0 #	0 #	0 #	0 #	0 #	0 #
50 µ/kg	0 #	0 #	0 #	0 #	0 #	0 #
100 µ/kg	0 #	0 #	0 #	0 #	0 #	0 #

Colitis severity was assessed in UT mice and mice treated with DSS for 4 days, followed by 3 days of daily oral gavage (OG) of budesonide or vehicle (PBS) (without DSS treatment) (*n* = 7 per group). * denotes a significant difference from UT mice; # denotes a significant difference from DSS/vehicle-treated mice.

### Effect of treatment with budesonide and minocycline combinations on the severity of already established colitis

**FIGURE 4:**
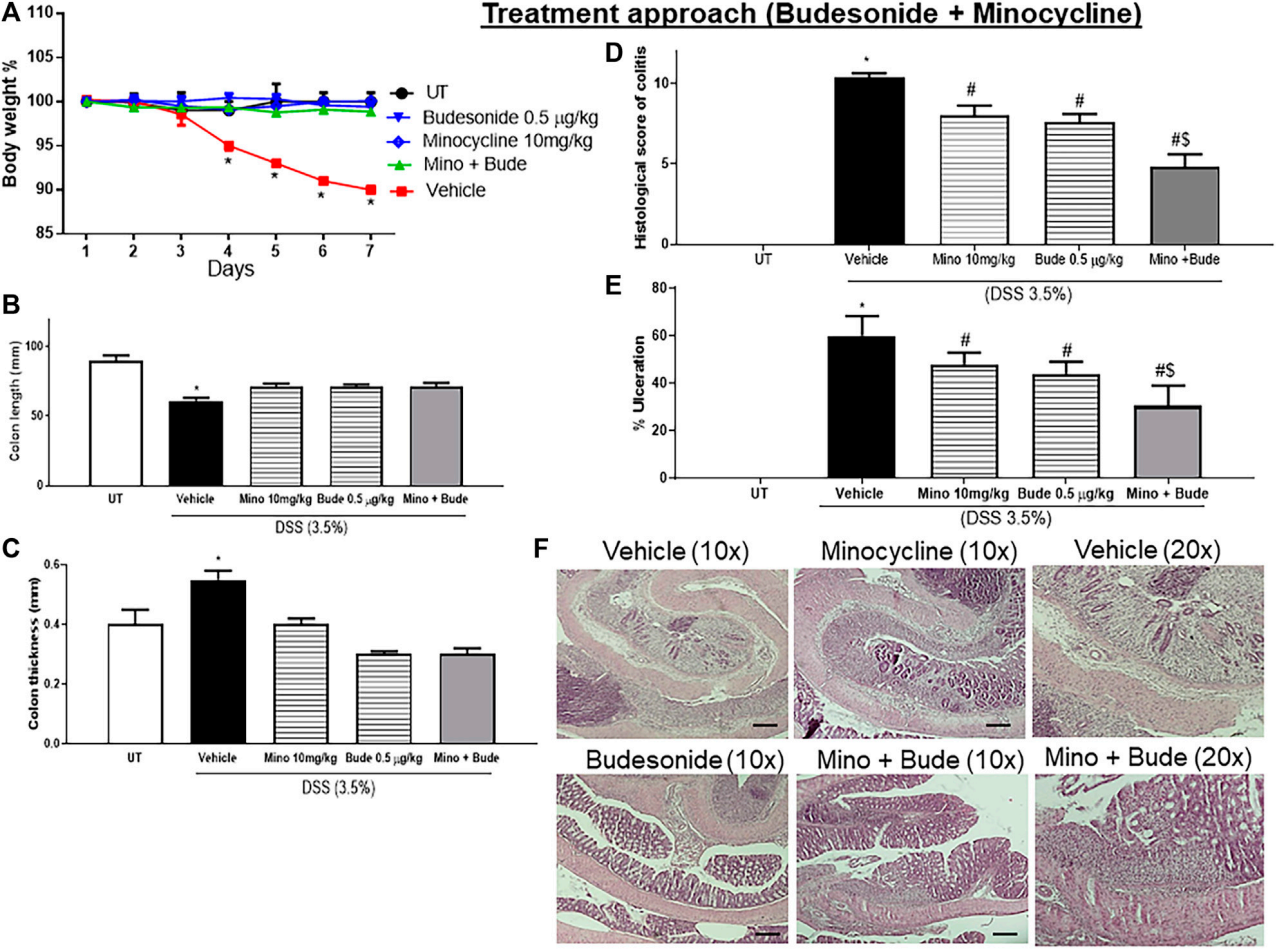
Effect of therapeutic treatment with minocycline, budesonide, or a combination regimen on colitis severity at the macroscopic and microscopic levels. **(A)** Percentage of body weight changes in DSS-treated mice receiving vehicle, minocycline (10 mg/kg), and budesonide (0.5 μg/kg), or untreated (UT) mice. Colon length **(B)**, thickness **(C)**, histological assessment of colitis severity **(D)**, and the percentage of ulceration in the whole colon section **(E)** were determined in mice receiving DSS and either vehicle (solid bars), monotherapy (hatched bars), or a combination regimen (gray bars), and in the UT healthy mice (open bars). Histobars represent means ± SEM for five mice in each group. Asterisks denote a significant difference from UT mice with *p* < 0.05 (*), # denotes a significant difference from DSS/vehicle-treated mice, and $ denotes a significant difference from monotherapy with *p* < 0.05. **(F)** Illustration of colon sections taken from various groups (×10 and ×20 magnifications; bars represent 150 μm).

Sub-optimal doses of minocycline and budesonide, 10 mg/kg minocycline and 0.5 μg/kg budesonide, were used to study whether there is a synergistic effect between the two drugs in reducing symptoms of colitis in mice. Treatment with minocycline, budesonide, or their combination reversed the DSS-induced drop in body weight (*p* < 0.05; Figure 4A), decrease in colon length (*p* < 0.05; Figure 4B), and increase in colon thickness (*p* < 0.05; Figure 4C). Treatment with individual drugs significantly reduced the DSS-induced edema, erythema, diarrhea, blood in stool, anorectal bleeding, and adhesions, but most importantly, the combination regimen completely reversed all these DSS-induced symptoms (Table 5). Treatment with minocycline or budesonide alone significantly reduced the DSS-induced histological score of colitis and percentage of ulceration, but most importantly, the combination regimen produced a more robust and significant reduction of these parameters than monotherapy (*p* < 0.05; Figures 4D, E). Figure 4F shows examples of colon sections taken from different treatment groups. Treatment with individual drugs reduced the various inflammatory parameters, but the combination regimen produced a higher degree of these parameters.

**TABLE 5:** Effect of therapeutic treatment with minocycline, budesonide, or a combination regimen on the macroscopic scores of colitis severity.

Parameter (%)	Edema	Erythema	Diarrhea	Blood in stool	Anorectal bleeding	Adhesion
UT	0	0	0	0	0	0
Vehicle	100 *	100 *	100 *	40 *	100 *	100 *
Mino 10 mg/kg	100 *	0 #	0 #	0 #	0 #	40 *#
Bud 0.5 µg/kg	40 *#	0 #	0 #	0 #	0 #	20 *#
Mino + Bud	0 #$	0 #	0 #	0 #	0 #	0 #$

Colitis severity was assessed in UT mice and mice treated with DSS for 4 days, followed by 3 days of daily i. p administration of minocycline, OG of budesonide, a combination regimen or vehicle (PBS) (without DSS treatment) (*n* = 5 per group). * denotes a significant difference from UT mice, # denotes a significant difference from DSS/vehicle-treated mice, and $ denotes a significant difference from monotherapy.

### Effect of treatment with methyl prednisolone on the severity of already established colitis

**FIGURE 5:**
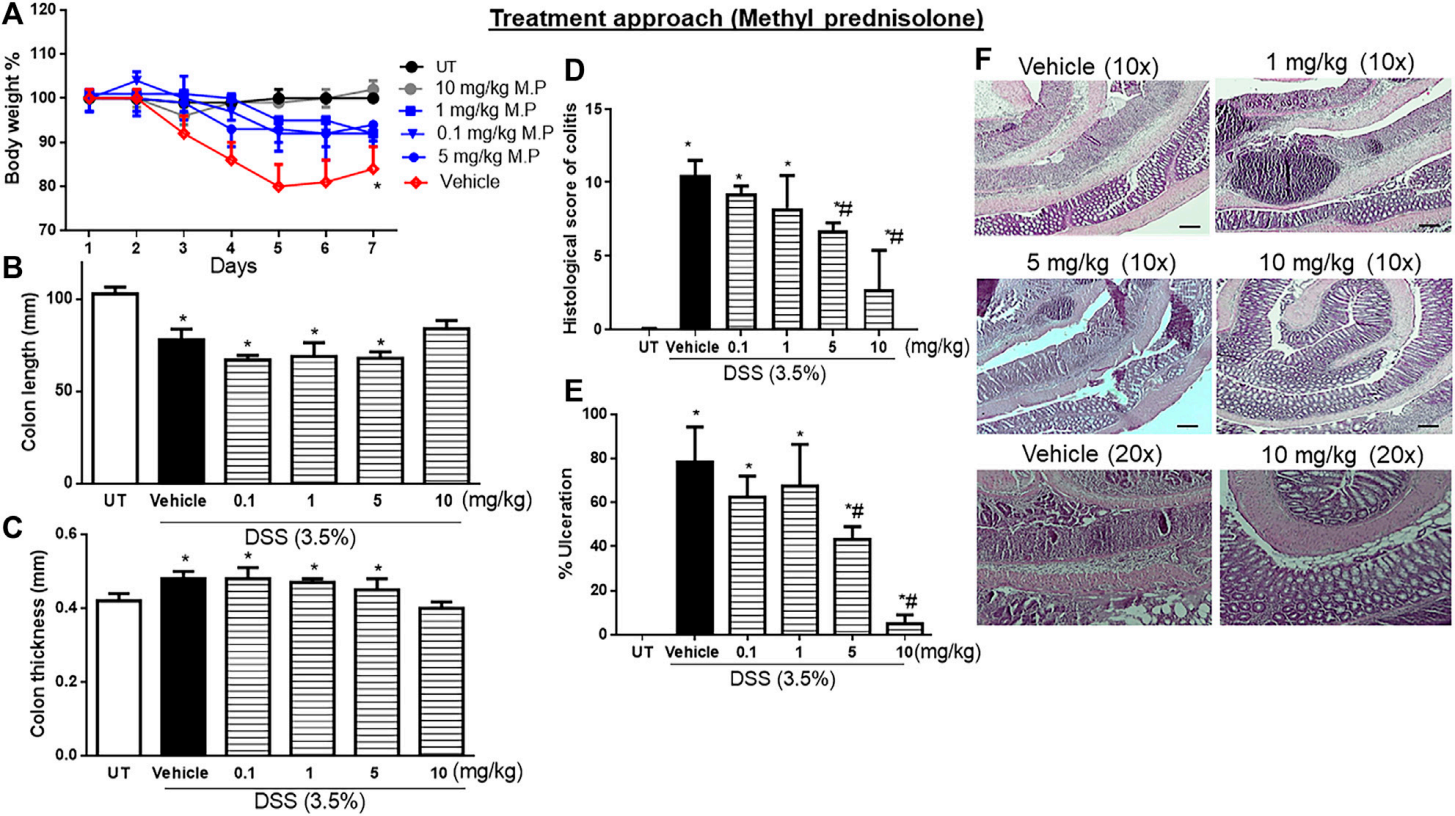
Effect of therapeutic treatment with methylprednisolone on colitis severity at the macroscopic and microscopic levels. **(A)** Percentage of body weight changes in DSS-treated mice receiving vehicle, and minocycline-treated mice at various doses compared to untreated mice. Colon length **(B)**, thickness **(C)**, histological assessment of colitis severity **(D)**, and the percentage of ulceration in the whole colon section **(E)** were determined in mice receiving DSS and either vehicle (solid bars) or various doses of minocycline (hatched bars) and in the UT healthy mice (open bars). Histobars represent means ± SEM for four mice in each group. Asterisks denote a significant difference from UT mice with *p* < 0.05 (*), and # denotes a significant difference from DSS/vehicle-treated mice with *p* < 0.05. **(F)** Illustration of colon sections taken from various groups (×10 and ×20 magnifications; bars represent 150 μm).

Treatment with another corticosteroid, methylprednisolone, at doses of 0.1–5 mg/kg partially reversed both DSS-induced drop in body weight, while a higher dose of 10 mg/kg restored the body weight to levels similar to those of UT mice (Figure 5A). Treatment with methylprednisolone at 10 mg/kg reversed both the DSS-induced decrease in colon length (*p* < 0.05; Figure 5B) and the increase in colon thickness (*p* < 0.05; Figure 5C). Methylprednisolone treatment (at doses of 0.1–5 mg/kg) significantly decreased the DSS-induced edema, erythema, diarrhea, blood in stool, anorectal bleeding, and adhesions, and these signs of inflammation were absent at a higher methylprednisolone dose of 10 mg/kg (Table 6). Treatment with methyl prednisolone at doses of 5–10 mg/kg significantly reduced the DSS-induced histological score of colitis and % of ulceration Figure 5D, E. Figure 5F shows examples of colon sections taken from different treatment groups. The various inflammatory parameters were significantly improved with methylprednisolone treatment, and the higher dose of 10 mg/kg eliminated the signs of inflammation.

**TABLE 6:** Effect of therapeutic treatment with methylprednisolone on the macroscopic scores of colitis severity.

Parameter (%)	Edema	Erythema	Diarrhea	Blood in stool	Anorectal bleeding	Adhesion
UT	0	0	0	0	0	0
Vehicle	100 *	100 *	100 *	40 *	100 *	100 *
Mino 10 mg/kg	100 *	0 #	0 #	0 #	0 #	40 *#
Bud 0.5 µg/kg	40 *#	0 #	0 #	0 #	0 #	20 *#
Mino + Bud 5 mg/kg	0 #$	0 #	0 #	0 #	0 #	0 #$

Colitis severity was assessed in UT mice and mice treated with DSS for 4 days, followed by 3 days of daily i. p administration of methylprednisolone or vehicle (PBS) (without DSS treatment) (*n* = 4 per group). * denotes a significant difference from UT mice; # denotes a significant difference from DSS/vehicle-treated mice.

### Effect of treatment with methylprednisolone and minocycline combinations on the severity of already established colitis

**FIGURE 6:**
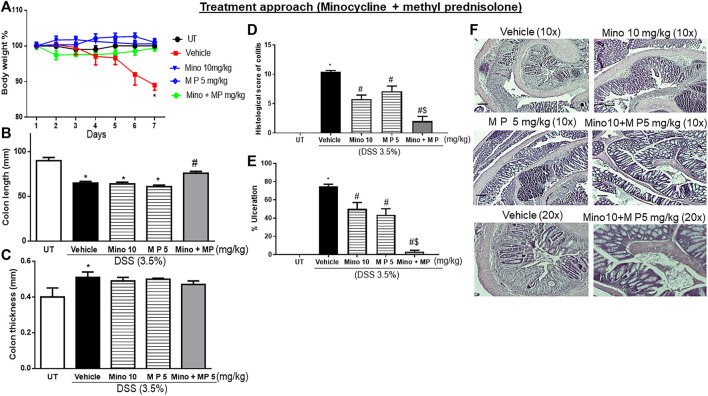
Effect of therapeutic treatment with minocycline, methylprednisolone, or a combination regimen on colitis severity at the macroscopic and microscopic levels. **(A)** Percentage of body weight changes in DSS-treated mice receiving vehicle, minocycline (10 mg/kg), methylprednisolone (5 mg/kg), and a combination regimen, or untreated (UT) mice. Colon length **(B)**, thickness **(C)**, histological assessment of colitis severity **(D)**, and the percentage of ulceration in the whole colon section **(E)** were determined in mice receiving DSS and either vehicle (solid bars), monotherapy (hatched bars), and a combination regimen (gray bar), or in the UT healthy mice (open bars). Histobars represent means ± SEM for five mice in each group. Asterisks denote a significant difference from UT mice with *p* < 0.05 (*), # denotes a significant difference from DSS/vehicle-treated mice, and $ denotes a significant difference from monotherapy with *p* < 0.05. **(F)** Illustration of colon sections taken from various groups (×10 and ×20 magnifications; bars represent 150 μm).

Sub-optimal doses of minocycline and methylprednisolone, 10 mg/kg minocycline and 5 mg/kg methyl prednisolone, were used to study whether there is a synergistic effect between the two drugs in reducing symptoms of colitis in mice. Treatment with the individual drugs or their combination reversed the DSS-induced drop in body weight (*p* < 0.05; Figure 6A), while only the combination reversed the DSS-induced decrease in colon length (*p* < 0.05; Figure 6B) and increase in colon thickness (*p* < 0.05; Figure 6C). Treatment with individual drugs significantly reduced DSS-induced edema, erythema, diarrhea, blood in stool, anorectal bleeding, and adhesions, but most importantly, the combination regimen completely reversed all these DSS-induced symptoms (Table 7). Treatment with minocycline or methylprednisolone alone significantly reduced the DSS-induced histological score of colitis and percentage of ulceration, but most importantly, the combination regimen produced a more robust and significant reduction of these parameters than monotherapy (*p* < 0.05; Figures 6D, E). Figure 6F shows examples of colon sections taken from different treatment groups. The various inflammatory parameters were significantly improved with methylprednisolone or minocycline treatment, but the combination regimen completely eliminated these signs of inflammation.

**TABLE 7:** Effect of therapeutic treatment with minocycline, methylprednisolone, or a combination regimen on the macroscopic scores of colitis severity.

Parameter (%)	Edema	Erythema	Diarrhea	Blood in stool	Anorectal bleeding	Adhesion
UT	0	0	0	0	0	0
Vehicle	100 *	75 *	92 *	33 *	66 *	100 *
0.05 mg/kg i.p	43 *#	0 #	0 #	0 #	14 #	57 *#
0.1 mg/kg i.p	0 #	0 #	0 #	0 #	14 #	57 *#
1 mg/kg i.p	0 #	0 #	0 #	0 #	0 #	57 *#
3 mg/kg i.p	0 #	0 #	0 #	0 #	0 #	33 *#
6 mg/kg i.p	0 #	0 #	0 #	0 #	0 #	0 #
3 mg/kg OG	0 #	0 #	0 #	0 #	0 #	0 #
6 mg/kg OG	0 #	0 #	0 #	0 #	0 #	0 #

Colitis severity was assessed in UT mice and mice treated with DSS for 4 days, followed by 3 days of daily i. p administration of minocycline, methylprednisolone, combination regimen, or vehicle (PBS) (without DSS treatment) (*n* = 5 per group). * denotes a significant difference from UT mice, # denotes a significant difference from DSS/vehicle-treated mice, and $ denotes a significant difference from monotherapy.

### Effect of treatment with dexamethasone on the severity of already established colitis

Treatment with various doses of dexamethasone (i.p. and oral gavage routes), another corticosteroid, significantly reduced signs of colitis at the gross but not at the histological level (Supplementary Figure S1; Supplementary Table S1). Therefore, we did not use dexamethasone in the combination regimen with minocycline.

### Comparison of the effect of treatment with minocycline plus budesonide combination versus minocycline plus methylprednisolone combination on the severity of already established colitis

The sub-optimal doses and the route of administration for the two corticosteroids in combination with minocycline are different (budesonide = 0.5 μg/kg oral gavage and methyl prednisolone = 5 mg/kg intraperitoneal injection). The degree of reduction in the histological score of inflammation in the combination regimen vs. monotherapy was more profound with methyl prednisolone (Figure 6D; approximately 69%) compared to budesonide (Figure 4D; approximately 38%). For this, the combination regimen of minocycline plus methyl prednisolone was used for the subsequent experiments to determine the effects of treatment on the colonic expression/activity profiles of various pro-inflammatory and pro-apoptotic molecules.

### Effect of treatment with minocycline, methylprednisolone, or a combination regimen on the colonic expression/activity of granulocyte markers, pro-inflammatory molecules, and molecules involved in the apoptotic machinery

**FIGURE 7:**
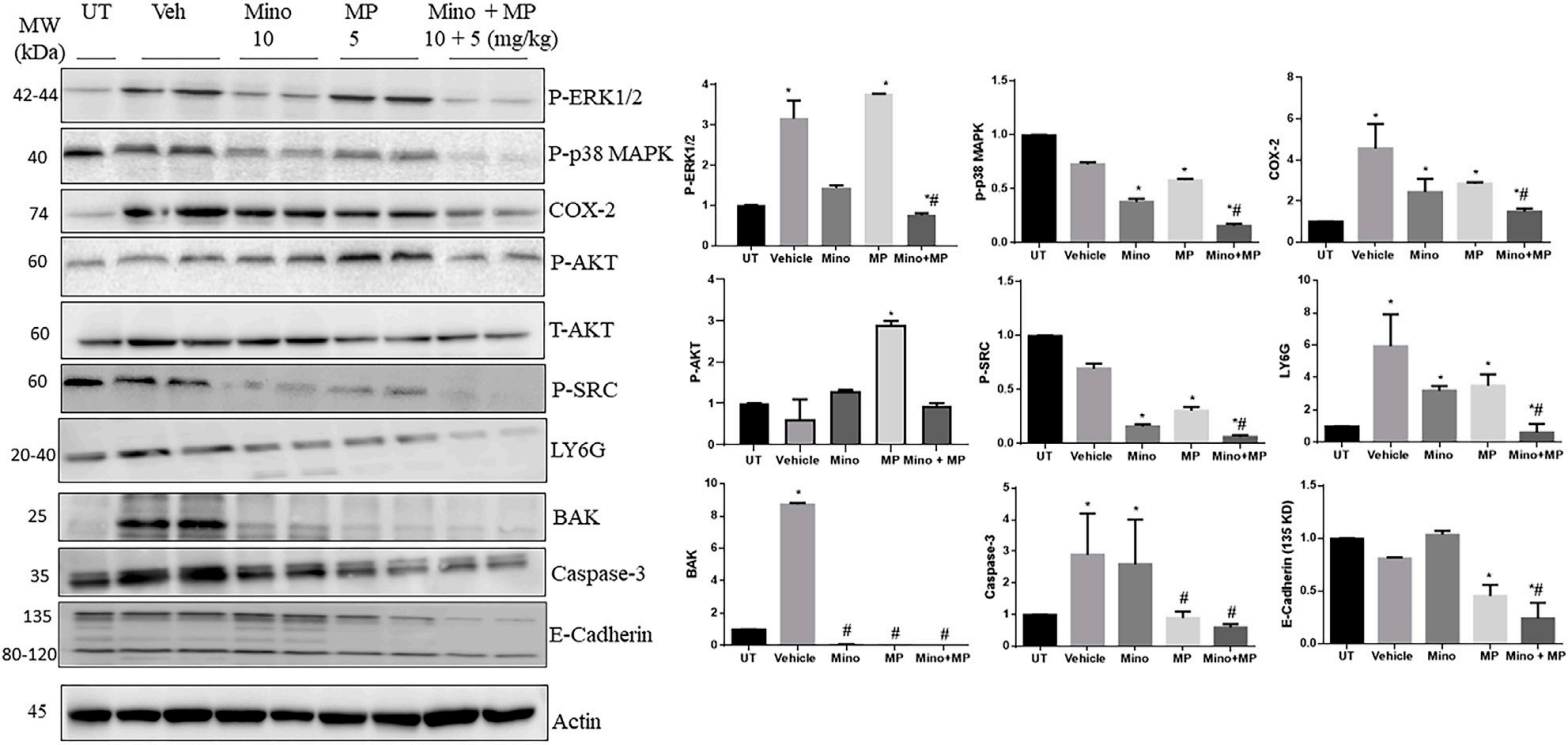
Effect of therapeutic treatment with minocycline, methylprednisolone, or a combination regimen on the colonic expression/activity of granulocyte markers, pro-inflammatory molecules, and molecules involved in the apoptotic machinery. The colonic expression/phosphorylation levels of various proteins were determined using Western blotting analysis. The blots represent one of three similar experiments. The densitometric analysis of the blots (arbitrary units) is also shown for each molecule. The *y*-axis represents the fold change between the tested groups (UT set as 1).

Next, we used Western blot analysis to determine the possible mechanism by which these drugs reduce DSS-induced inflammation in the colon of mice (Figure 7). Administration of DSS induced a significant increase in phospho-ERK1/2, the pro-inflammatory enzyme COX-2, the granulocyte marker LY6G, BAK, and caspase 3 compared to UT mice, which were all consistently reduced by treatment with the minocycline and methyl prednisolone combination regimen, while the individual drugs had variable effects. There were no differences in the levels of phospho-p38 MAPK, phospho-AKT, phospho-SRC, and E-cadherin in the colon between DSS-treated and UT mice. However, treatment with the combination regimen significantly reduced the phosphorylation of p38 MAPK, AKT, and SRC when compared to UT, vehicle, and monotherapy. The total AKT level was not modulated in all the tested groups. Finally, the combination regimen reduced the expression of E-cadherin when compared to UT, vehicle, and monotherapy.

### Effect of treatment with minocycline and methylprednisolone combination regimens on the blood and colonic gene expression of pro-/anti-inflammatory cytokines

A pilot study showed that the relative expression of the genes for the cytokines (*Il1b*, *Tnf*, and *Il10*) in the colon exhibited a trend to increase at day 3 after DSS/vehicle treatment, returning to UT levels by day 7 (Supplementary Figure S2).

**FIGURE 8:**
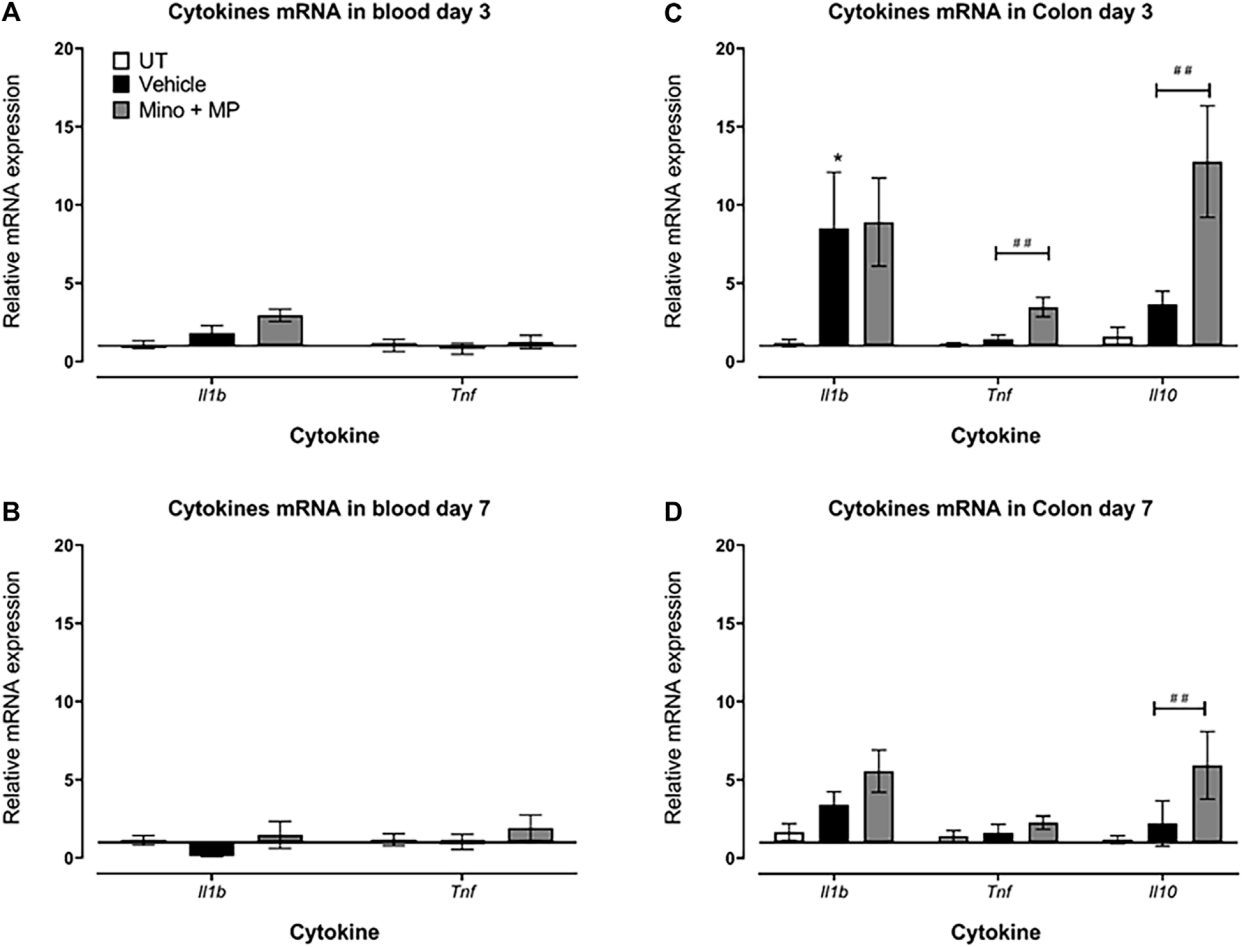
Effect of therapeutic treatment with the minocycline and methylprednisolone combination (MMP) regimen on the blood and colonic gene expression of pro- and anti-inflammatory cytokines The relative mRNA expression of the cytokines *Il1b* and *Tnf* in the blood both at **(A)** day 3 and **(B)** day 7 in untreated, DSS/vehicle (V)-treated, and DSS/minocycline plus methyl prednisolone (Mino + MP)-treated mice. Histobars represent the median plus the interquartile range of 3–5 mice in each group. The relative mRNA expression of the cytokines *Il1b*, *Tnf*, and *Il10* in the colon both at **(C)** days 3 and **(D)** 7 in UT, V-treated, and Mino + MP-treated mice. Histobars represent the median plus the interquartile range of 7–19 mice in each group. * denotes a significant difference from UT mice (*p* < 0.05); ## denotes a significant difference from DSS/vehicle-treated mice (*p* < 0.01).

DSS/vehicle treatment did not alter the expression of the genes of the cytokines *Il1b* and *Tnf* in the blood at days 3 and 7 after the first administration of DSS compared to the UT group (Figures 8A, B). *Il10* transcripts were lowly expressed in the blood of both control untreated (three out of the five samples undetected) or DSS/vehicle-treated mice (four out of the five samples undetected), and therefore, relative gene expression could not be calculated, while its transcripts were detected in all samples of the colon.

There was a significant increase in *Il1b* transcript levels in the colon of DSS/vehicle-treated mice compared to UT mice on day 3, while there were no differences in the expression of *Tnf* and *Il10* transcripts between the DSS/vehicle-treated mice and the UT mice (Figure 8C). Treatment with the combination regimen of minocycline and methylprednisolone had no significant effects on the DSS-induced changes in *Il1b* transcript expression but increased the *Tnf* and *Il10* transcript levels on day 3 (Figure 8C). On day 7, there were no differences in the levels of the transcripts of *Il1b*, *Tnf*, and *Il10* between the DSS/vehicle-treated mice and UT mice (Figure 8D). Treatment with minocycline and methylprednisolone had no significant effects on the DSS-induced changes in *Il1b* and *Tnf* transcript expression but increased the *Il10* transcript levels on day 7 (Figure 8D).

## Discussion

In the current study, minocycline treatment was effective in reducing colitis severity in mice and produced a synergistic effect with budesonide and methylprednisolone in alleviating active colitis. Lower doses of budesonide or methyl prednisolone, when used in combination with minocycline, produced similar anti-inflammatory effects as higher doses of budesonide or methyl prednisolone when used alone. This is of clinical importance in terms of targeting more than one arm of the pathological features of IBD, the complex inflammatory response and the bacterial components, as well as in terms of reducing the dose-dependent side effect profile of corticosteroids and the total cost of the treatment.

Currently, there are few studies that have described the role of minocycline in animal models of colitis. In one report, minocycline treatment significantly reduced colitis severity in the TNBS model by reducing the colonic expression levels of molecules involved in the inflammatory process such as TNF-α, IL-1β, iNOS, IL-17, IL-6, monocyte chemotactic protein-1 (MCP-1), cytokine-induced neutrophil chemoattractant-1 (CINC-1), and intercellular adhesion molecule 1 (ICAM-1) (Garrido-Mesa et al., 2013b). Minocycline treatment also resulted in increased colonic glutathione, mucin-2 (MUC-2), and trefoil factor-3 (TFF-3) levels. Another report showed that minocycline reduced the colonic expression levels of TNF-α, IL-1β, IL-6, matrix metalloproteinase (MMP)-2, -3, -9, and -13, and the serum level of nitric oxide (Huang et al., 2009). In addition, a combination regimen of minocycline and the probiotic *Escherichia coli* Nissle 1917 (5 × 10^8^ CFU/day; p. o, using the DSS model) resulted in synergistic anti-inflammatory effects. This was in part due to the reduction in the expression of IL-1β, TNF-α, IL-2, MIP-2, MCP-1, ICAM-1, iNOS, and MMP-9 (Garrido-Mesa et al., 2011). Furthermore, the combination regimen restored intestine integrity by upregulating the expression levels of MUC-3 and the TJ protein zonula occludens-1 (ZO-1). In the current study, both prophylactic and therapeutic treatment with minocycline significantly reduced colitis severity at both gross and histological levels (Figures 1, 2; Table 2, 3).

Dysregulation in the apoptotic machinery has been linked to IBD pathogenesis. Enhanced apoptotic colonic epithelial cells have been observed to have active UC (Boughton-Smith et al., 1993; Middleton et al., 1993; Lundberg et al., 1994). The colonic levels of various caspases were found to be increased in various animal models of colitis. For example, caspase-3 (Anselmi et al., 2015; Tang et al., 2015; Qian et al., 2016), -7 (Anselmi et al., 2015), and -12 (Crespo et al., 2012), and the cleaved forms of caspase-1 (Liu et al., 2016; Marquez-Flores et al., 2016), -12, and -7 (Arumugam et al., 2015) significantly increased post-colitis induction. Our data that demonstrated enhanced BAK and caspase-3 expression in the descending colon of mice subjected to DSS agree with these studies.

Neutrophils play an important role in IBD pathogenesis through enhanced recruitment and/or delayed apoptosis at the inflammatory site (Hirota et al., 2011), which leads to enhanced oxidative stress activity, leading to tissue damage (Yamada and Grisham, 1991; Gayle et al., 2000). Thus, depleting circulating neutrophils (Natsui et al., 1997; Qualls et al., 2006) or the administration of antioxidants significantly reduced colitis severity (Yamada and Grisham, 1991). In the current study, there was increased colonic expression of the granulocyte marker LY6G in DSS-treated mice, which was significantly decreased with minocycline and methylprednisolone treatment (Figure 7). However, more detailed cellular studies are required to fully understand the molecular mechanisms of the disease and the drugs used to treat it.

The contribution of various pro-inflammatory signaling molecules such as COX-2, SRC, and ERK1/2 in IBD pathogenesis is well defined. For example, enhanced colonic activity of the MAPK pathway has been observed in IBD patients (Hommes et al., 2002; Jin et al., 2015), and the pro-apoptotic effects of pro-inflammatory cytokines such as TNF-α were mediated through enhanced MEK/ERK activation (Bai et al., 2015). In addition, enhanced COX-2 and SRC activity was observed in DSS-treated mice, which contributed to colitis pathogenesis (Tolstanova et al., 1999; Seok Yang et al., 2012). We observed enhanced expression/activity profiles of these signaling molecules in the colonic tissues of DSS-treated mice, which were reduced by minocycline and methylprednisolone treatment at different degrees (Figure 7). Furthermore, T_H_-1-mediated cytokines such as IL-1β and TNF-α contribute to colitis pathogenesis and are important therapeutic targets for IBD management (Stevens et al., 1992; Reinecker et al., 1993; Elsässer-et al., 1994; Woywodt et al., 1994; Dionne et al., 1997). These cytokines enhance the release of other cytokines and chemokines, and reactive oxygen species contribute to the severity of colitis (Papadakis and Targan, 2000). In the current study, the transcript levels of the pro-inflammatory cytokine *Il1b* were significantly increased in the colon of the colitis model at day 3 and returned to normal levels by day 7 (Figure 8). There were no changes in the expression of the transcript levels of the cytokines in the blood, suggesting a more localized inflammation reaction in the colon of the mice. More interestingly, both the prophylactic and therapeutic treatment with minocycline and methylprednisolone had no significant effect on the levels of the transcripts of the pro-inflammatory cytokine *Il1b* but increased the transcript levels of the anti-inflammatory cytokine *Il10*, suggesting that in terms of cytokines, the treatment favored resolution of inflammation by increasing the levels of the anti-inflammatory cytokine.

Various corticosteroids are currently used for the induction (and sometimes for maintenance) of disease activity. Their usage is limited due to their various dose-dependent side effects, such as an increase in blood glucose levels and an increased risk of osteoporosis and infections (Mishra et al., 2020). In this study, using a low dose of either budesonide (Figure 4) or methyl prednisolone (Figure 6) in combination with a low dose of minocycline (10 mg/kg) reduced colitis severity dramatically more than either drug alone. These results are of clinical importance as they target more than one arm of the inflammatory process and reduce the effective dose of steroids to half, thus reducing the chance of side effects.

In conclusion, we confirmed that minocycline is effective in reducing colitis severity in mice and synergizes with corticosteroids (cutting their doses by half) to reduce their dose-dependent side effects and treatment cost. This was achieved by modulating the expression/activity profiles of various pro- and anti-inflammatory signaling molecules, cytokines, and molecules involved in the apoptotic machinery.

## Data Availability

The original contributions presented in the study are included in the article/Supplementary Material; further inquiries can be directed to the corresponding author.
