# Supplementary material for: Minocycline synergizes with corticosteroids in reducing colitis severity in mice via the modulation of pro-inflammatory molecules
Source: Front Pharmacol. 2023 Nov 16;14:1252174. doi: 10.3389/fphar.2023.1252174 (PMC10687282; doi:10.3389/fphar.2023.1252174)
Supplement: Supplementary file 1 [file Table1.docx]

**Supplementary table 1. Effect of dexamethasone on colitis severity at macroscopic level using the treatment approach**

| Parameter (%) | Edema | Erythema | Diarrhea | Blood in stool | Anorectal bleeding | Adhesion |
| --- | --- | --- | --- | --- | --- | --- |
| UT | 0 | 0 | 0 | 0 | 0 | 0 |
| Vehicle | 100 * | 75 * | 92 * | 33 * | 66 * | 100 * |
| 0.05 mg/kg i.p | 43 *# | 0 # | 0 # | 0 # | 14 # | 57 *# |
| 0.1 mg/kg i.p | 0 # | 0 # | 0 # | 0 # | 14 # | 57 *# |
| 1 mg/kg i.p | 0 # | 0 # | 0 # | 0 # | 0 # | 57 *# |
| 3 mg/kg i.p | 0 # | 0 # | 0 # | 0 # | 0 # | 33 *# |
| 6 mg/kg i.p | 0 # | 0 # | 0 # | 0 # | 0 # | 0 # |
| 3 mg/kg OG | 0 # | 0 # | 0 # | 0 # | 0 # | 0 # |
| 6 mg/kg OG | 0 # | 0 # | 0 # | 0 # | 0 # | 0 # |
