# Supplementary figures and images for: Minocycline synergizes with corticosteroids in reducing colitis severity in mice via the modulation of pro-inflammatory molecules
Source: Front Pharmacol. 2023 Nov 16;14:1252174. doi: 10.3389/fphar.2023.1252174 (PMC10687282; doi:10.3389/fphar.2023.1252174)

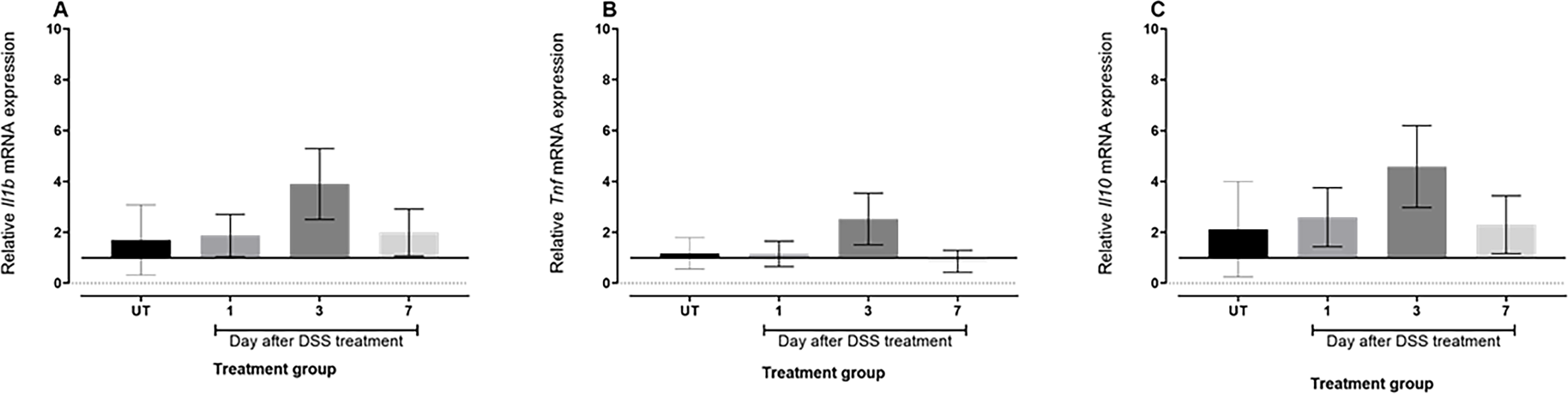

Supplement: Supplementary file 2 [file Image2.TIF]

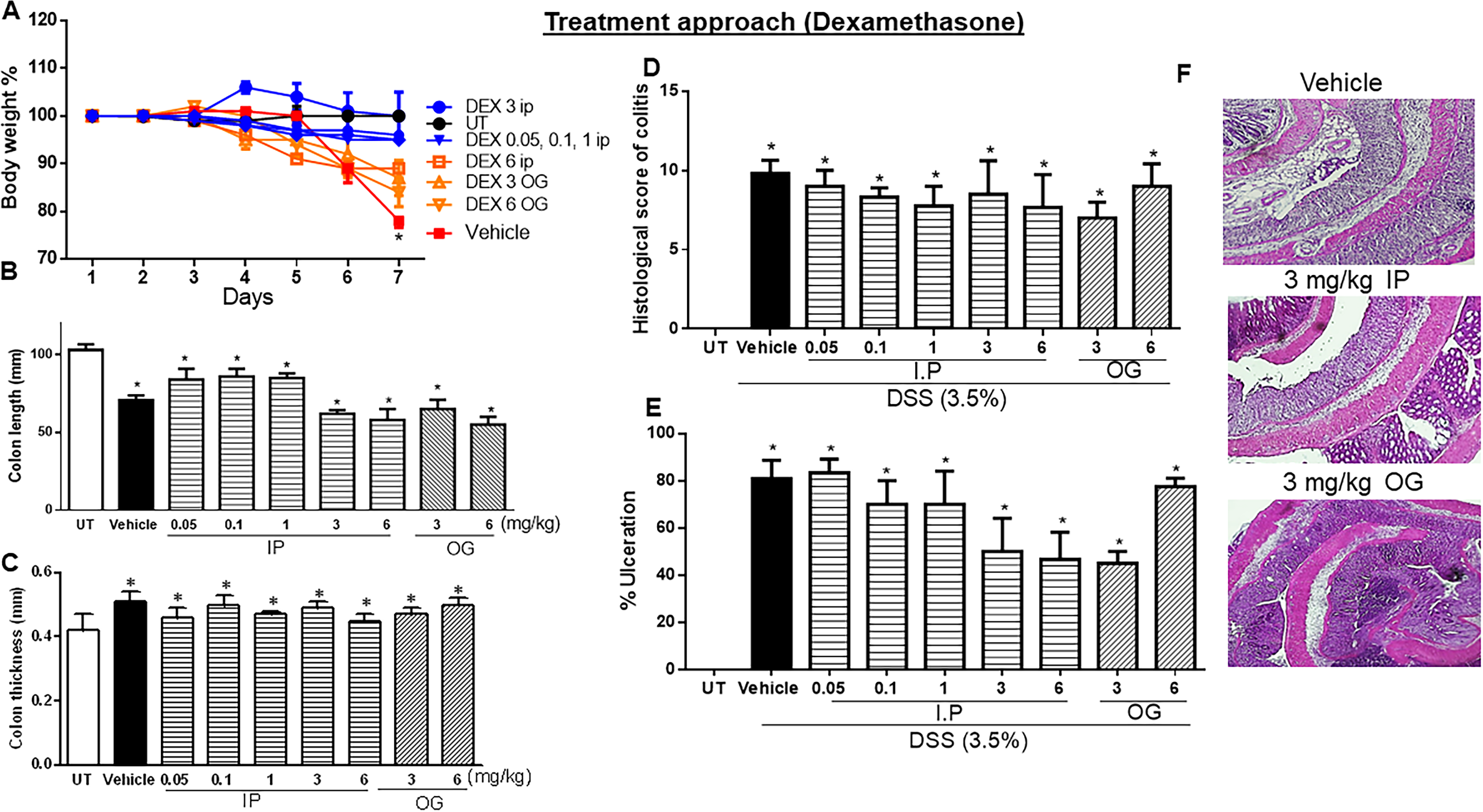

Supplement: Supplementary file 3 [file Image1.TIF]
